# Supplementary material for: Transcriptome-Based Analysis Reveals a Crucial Role of BxGPCR17454 in Low Temperature Response of Pine Wood Nematode (Bursaphelenchus xylophilus)
Source: Int J Mol Sci. 2019 Jun 14;20(12):2898. doi: 10.3390/ijms20122898 (PMC6628231; doi:10.3390/ijms20122898)
Supplement: Supplementary file 1 [file ijms-20-02898-s001.zip › ijms-492266 - supplementary/Supplementary Materials-Table S1,S2 and S5, S6, Figure S1, S2 and S3.pdf]

Supplementary Materials

Transcriptome-based Analysis Reveals a Crucial Role of *BxGPCR17454* in Low Temperature Response of Pine Wood Nematode (*Bursaphelenchus xylophilus*)

Table S1. Primers used in qRT-PCR validation for RNA sequencing data

Gene ID	Primer Names	Primer Sequences 5'-3'
BXY_0069900	BXY_0069900-F	AATGCAACGAGGCTCCGAGATG
	BXY_0069900-R	AGGCTGGATGGCGTCAATCAATC
BXY_0262500	BXY_0262500-F	GCGCCGAATTCCACATCAATGC
	BXY_0262500-R	CCATCGCCAGCACTGGACTTG
BXY_0413400	BXY_0413400-F	GTGACAGCCGACGAAGAAGAGTTC
	BXY_0413400-R	AAGGACCAGACCGAGCCGAATC
BXY_0508700	BXY_0508700-F	GGCGATCAGTTCAAGGAAGAGGTC
	BXY_0508700-R	AATTCAGCCGCAGCAACTCCAG
BXY_0596900	BXY_0596900-F	ATTGAGACCAGGGACAGGCA
	BXY_0596900-R	TCGGGCTCAGGGATACCTTC
BXY_0688500	BXY_0688500-F	GGTATCTGGACGACACCAAGACAC
	BXY_0688500-R	CCACGCTGAGATCCTTGAACGG
BXY_0794300	BXY_0794300-F	TGGCGGTCAGCGACATTGTAATG
	BXY_0794300-R	CGGCGATGGCTGTGAGTGTG
BXY_0799000	BXY_0799000-F	CGTTCCGTCACCGTATGCCTTG
	BXY_0799000-R	GCATTCTGATGGAGCAGCCGATC
BXY_0959300	BXY_0959300-F	CCTCGGAAGTTCAACGCCTCAC
	BXY_0959300-R	CGAACGTAATCCACTGCGGTCTC
BXY_1220600	BXY_1220600-F	GCCACGGCTCCTTCCTCCTC
	BXY_1220600-R	GGCAATCCTCGCATGAACGGTAG
BXY_1423800	BXY_1423800-F	CACAGGAAGACGCCTCTCCAATTC
	BXY_1423800-R	CGCTGCTCCACAATCCTCTTGAC
BXY_1722500	BXY_1722500-F	CGATTCAACTGGCTAAGCGTCCTG
	BXY_1722500-R	GCGATCTTGTTCCAAGGTGGTCTC
Bx28S	Bx28s-F	GTGCGTATTCAGCCTTCTGG
	Bx28s--R	AACCGAACACGCGACAATAG

Table S2. Primers used in transcript abundance analysis of 6 low-temperature-related *BxGPCRs*

Gene names	Gene ID	Primer Names	Primer sequences 5'-3'
BxGPCR13758	BXY_1375800	q-BXY_1375800-F	ATGACCATCGACCGATACCTCTCC
		q-BXY_1375800-R	ACACCGTGCGTTCATGTGGATC
BxGPCR16727	BXY_1672700	q-BXY_1672700-F	ATGTGGTGTTGGTGGCTGATGTC
		q-BXY_1672700-R	TCGCCGTGTAGTCCTGGTAGC
BxGPCR05932	BXY_0593200	q-BXY_0593200-F	CCGAGCCGTATGATGAACTTGGAG
		q-BXY_0593200-R	CAGAGCACAGCAGCAACATTCTTG
BxGPCR05210	BXY_0521000	q-BXY_0521000-F	TCGTTCCGACGGAGGATTCTGG
		q-BXY_0521000-R	ATCATTCTCAGCTCTGGCGTTGAC
BxGPCR13915	BXY_1391500	q-BXY_1391500-F	CATCTCGGTTGCCTTGAATCGAAC

		q-BXY_1391500-R	ATGGTCGTCACACTGTTGATGAGG
BxGPCR17454	BXY_1745400	q-BXY_1745400-F	TGTGGATGTGAGCCCGAGAA
		q-BXY_1745400-R	TCCCCACAACAAACTTGGGC
β-actin	*β-actin*	β-actin-F	TTGGCTGGCCGTGACTTGAC
		β-actin-R	GCGGTGGCCATCTCCTGTTC
Bx28S	Bx28S	Bx28s-F	GTGCGTATTCAGCCTTCTGG
		Bx28s--R	AACCGAACACGCGACAATAG

Table S5. Conserved domain analysis of BxGPCRs with NCBI Batch CD

**Query**	**Hit type**	**PSSM-ID**	**From**	**To**	**E-Value**	**Bitscore**	**Accession**	**Short name**	**Incomplete**	**Superfamily**
Q#1 - >BXY_1062600	superfamily	325116	55	252	1.49E-09	57.1604	cl11665	7TM_GPCR_Srh superfamily	N	-
Q#6 - >BXY_1684700	superfamily	313533	183	382	1.18E-28	113.849	cl12383	7TM_GPCR_Srt superfamily	N	-
Q#7 - >BXY_1107100	superfamily	251743	204	341	3.49E-06	48.0867	cl10600	Sre superfamily	N	-
Q#15 - >BXY_1163000	superfamily	328955	493	585	4.57E-08	55.5452	cl22924	7TM_GPCR_Srd superfamily	N	-
Q#15 - >BXY_1163000	superfamily	325116	374	550	3.48E-06	49.8416	cl11665	7TM_GPCR_Srh superfamily	N	-
Q#7 - >BXY_1107100	superfamily	325099	21	243	6.32E-09	56.5055	cl11607	7TM_GPCR_Srab superfamily	C	-
Q#3 - >BXY_0593200	superfamily	328955	50	336	1.80E-18	84.5274	cl22924	7TM_GPCR_Srd superfamily	-	-
Q#4 - >BXY_0521000	superfamily	328955	18	310	2.00E-19	87.233	cl22924	7TM_GPCR_Srd superfamily	-	-
Q#5 - >BXY_0255900	superfamily	328955	44	331	1.07E-19	87.609	cl22924	7TM_GPCR_Srd superfamily	-	-
Q#9 - >BXY_1375800	specific	320109	31	324	3.03E-45	157.41	cd14978	7tmA_FMRFamide_R-like	-	cl28897
Q#10 - >BXY_1391500	superfamily	328955	18	301	7.61E-16	76.4382	cl22924	7TM_GPCR_Srd superfamily	-	-
Q#11 - >BXY_1525400	superfamily	328955	1	252	1.05E-11	64.0196	cl22924	7TM_GPCR_Srd superfamily	-	-
Q#12 - >BXY_1672700	superfamily	333717	15	248	1.03E-07	52.6352	cl28897	7tm_GPCRs superfamily	-	-
Q#14 - >BXY_0078500	superfamily	328955	107	396	4.05E-17	81.0606	cl22924	7TM_GPCR_Srd superfamily	-	-

Q#15 - >BXY_1163000	superfamily	328955	626	923	1.83E-12	68.7342	cl22924	7TM_GPCR_Srd superfamily	-	-
Q#15 - >BXY_1163000	superfamily	328955	17	315	4.34E-05	46.3927	cl22924	7TM_GPCR_Srd superfamily	-	-
Q#16 - >BXY_1745400	specific	320109	25	319	2.58E-40	144.698	cd14978	7tmA_FMRFamide_R-like	-	cl28897

Table S6. selected homologous amino acid sequences from NCBI

NCBI Accession	Description	Organism
XP_024498819	G protein-coupled receptor, rhodopsin-like family and GPCR, rhodopsin-like, 7TM domain-containing protein	*Strongyloides ratti*
PDM65196	G protein-coupled receptor	*Pristionchus pacificus*
NP_506330	Serpentine receptor class delta-18	*Caenorhabditis elegans*
XP_003112745	CRE-SRD-16 protein	*Caenorhabditis remanei*
XP_002647618	C.briggsae CBR-SRD-17 protein	*Caenorhabditis briggsae*
NP_504331	Seven TM Receptor	*Caenorhabditis elegans*
NP_503891	Serpentine Receptor, class J	*Caenorhabditis elegans*
XP_003110316	CRE-STR-135 protein	*Caenorhabditis remanei*
XP_024501207	G protein-coupled receptor, rhodopsin-like family and GPCR, rhodopsin-like, 7TM domain-containing protein	*Strongyloides ratti*
XP_024503330	G protein-coupled receptor, rhodopsin-like family and GPCR, rhodopsin-like, 7TM domain-containing protein	*Strongyloides ratti*
CDJ81496	7TM GPCR domain containing protein	*Haemonchus contortus*
EPB75428	7 transmembrane receptor	*Ancylostoma ceylanicum*
KJH44787	7 transmembrane receptor, partial	*Dictyocaulus viviparus*
XP_024500519	G protein-coupled receptor, rhodopsin-like family and GPCR, rhodopsin-like, 7TM domain-containing protein	*Strongyloides ratti*
XP_024498605	GPCR, rhodopsin-like, 7TM domain-containing protein	*Strongyloides ratti*
KHJ88425	7 transmembrane receptor, partial	*Oesophagostomum dentatum*
XP_013292781	7 transmembrane receptor	*Necator americanus*

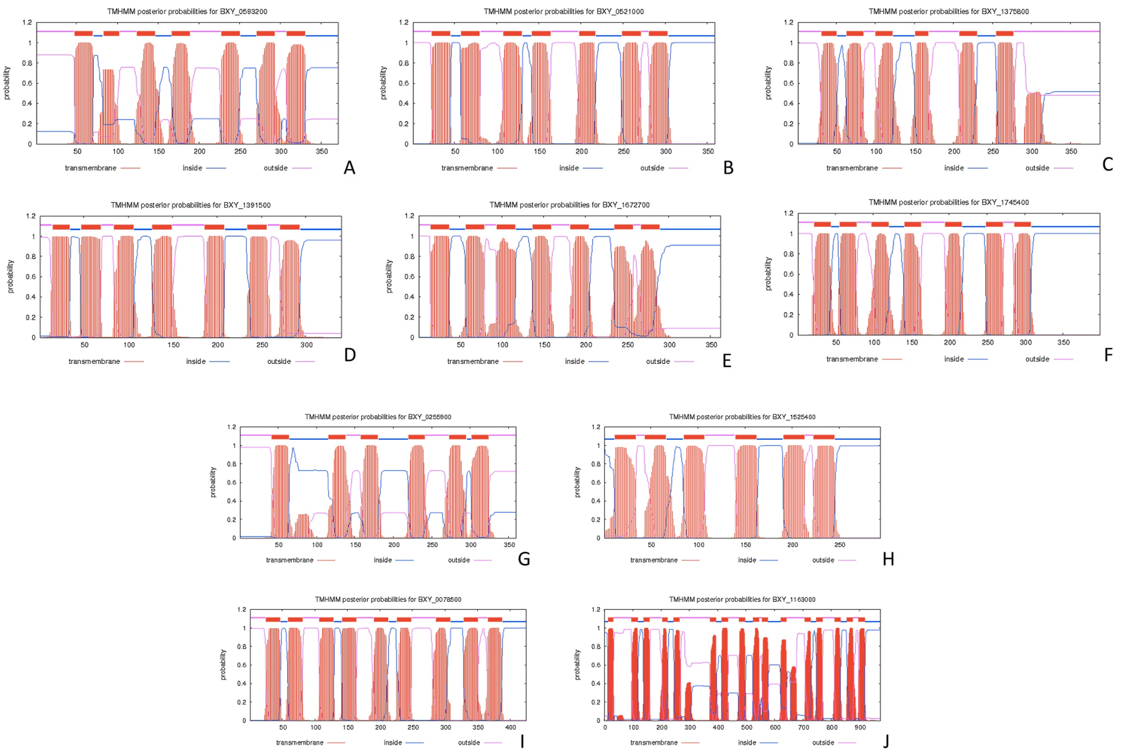

Figure S1. Transmembrane domains prediction of candidate BxGPCRs. **(A).** Transmembrane domains prediction of BXY_0593200. **(B).** Transmembrane domains prediction of BXY_0521000. **(C).** Transmembrane domains prediction of BXY_1375800. **(D).** Transmembrane domains of BXY_1391500. **(E).** Transmembrane domains prediction of BXY_1672700. **(F).** Transmembrane domains prediction of BXY_1745400. **(G).** Transmembrane domains prediction of BXY_0255900. **(H).** Transmembrane domains prediction of BXY_1525400. **(I).** Transmembrane domains prediction of BXY_0078500. **(J).** Transmembrane domains prediction of BXY_1163000.

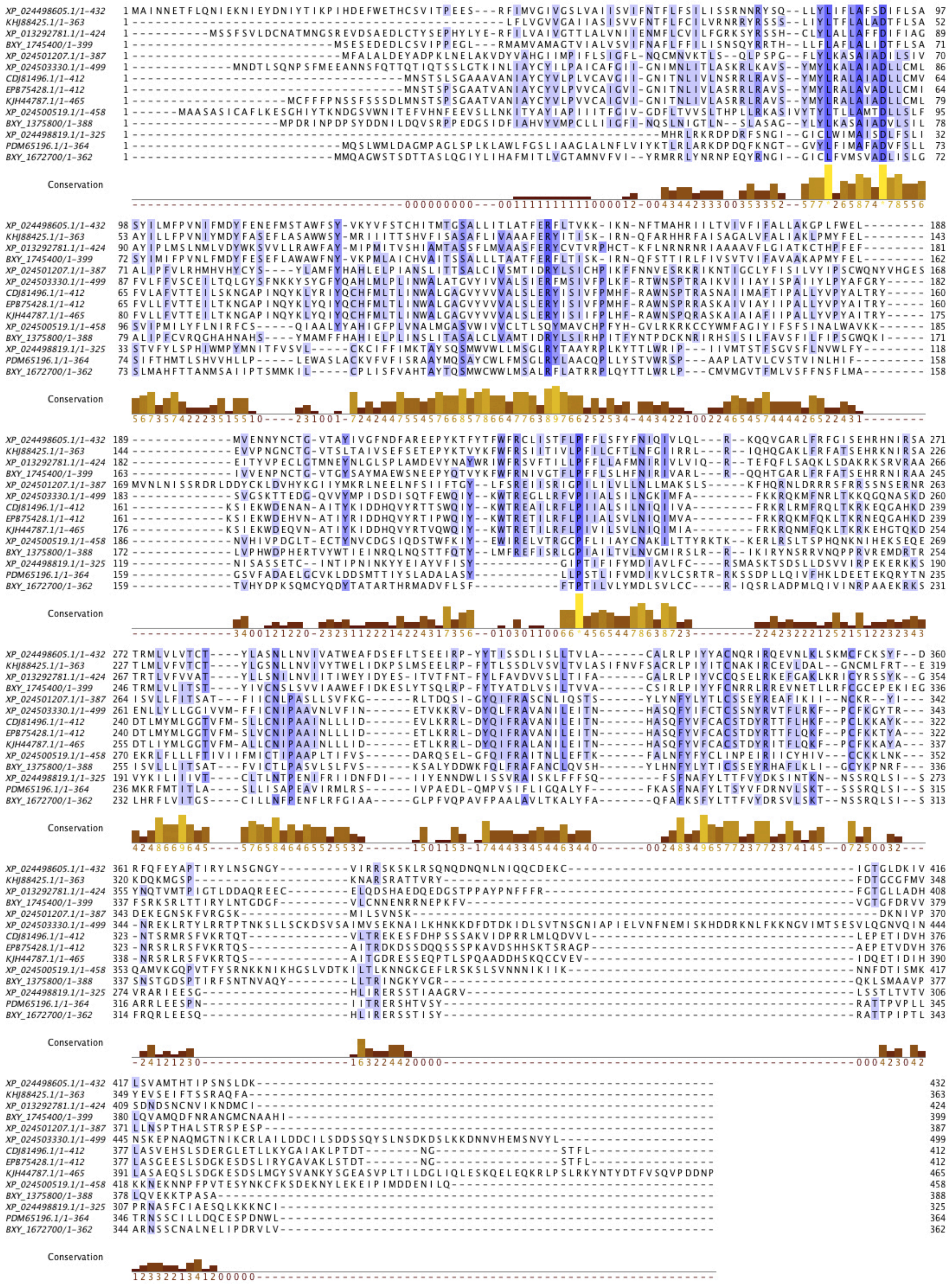

A

Figure S2. Cont.

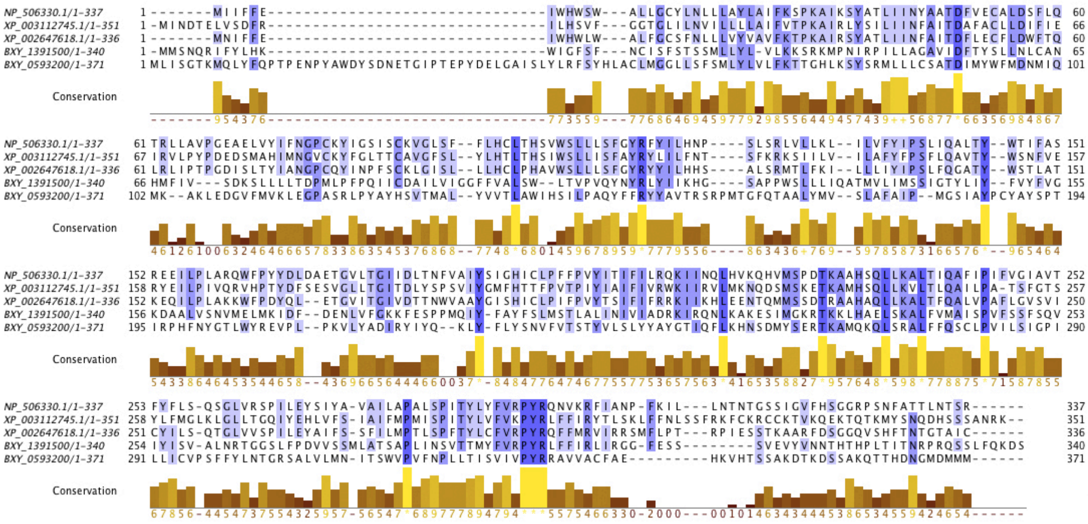

B

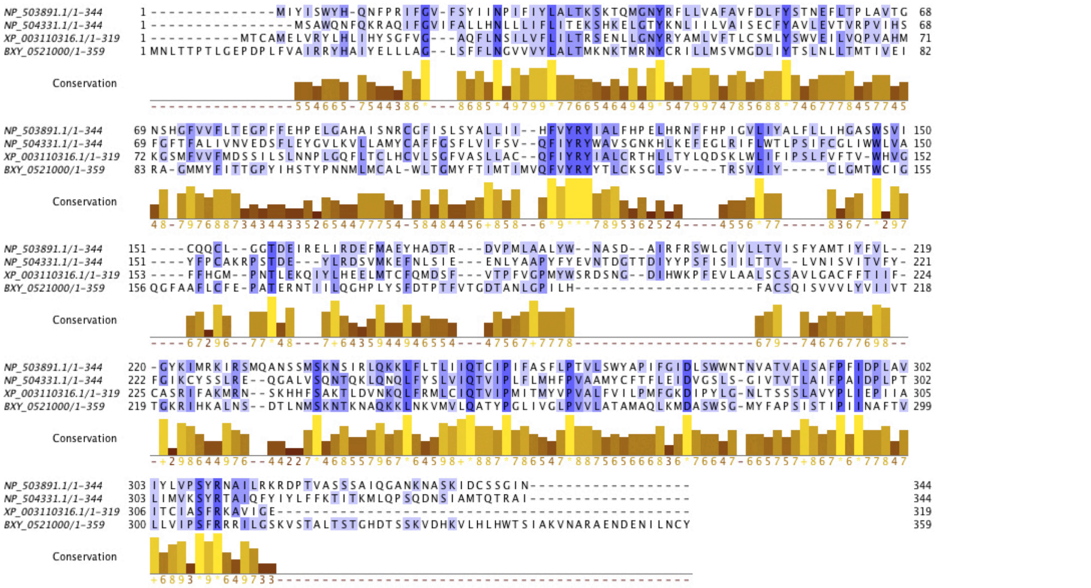

C

Figure S2. Alignment of the deduced amino acid sequences of the 6 predicted BxGPCRs and known GPCRs from NCBI. **(A).** Alignment of the deduced amino acid sequences of BXY_1745400, BXY_1375800 and BXY_1672700 along with known GPCRs from NCBI. **(B).** Alignment of the deduced amino acid sequences of BXY_0593200 and BXY_1391500 along with known GPCRs from NCBI. **(C).** Alignment of the deduced amino acid sequences of BXY_0521000 along with known GPCRs from NCBI.

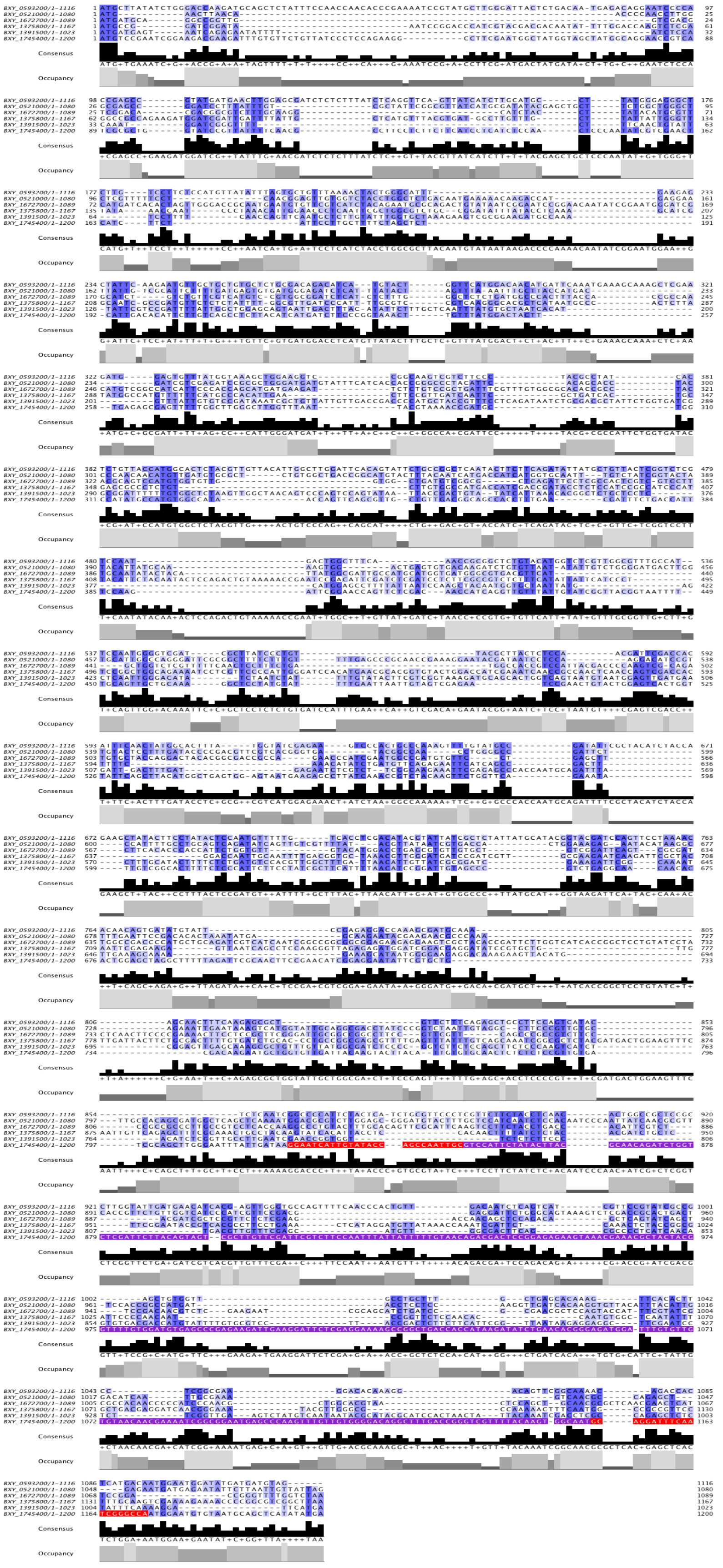

Figure S3. Alignment of 6 BxGPCRs nucleic sequences. The RNAi primer regions are coloured in red and dsRNA regions are coloured in purple.
